# Melanocytic Nevus in the External Auditory Canal with Keratin Accumulation

**DOI:** 10.1155/2021/5539286

**Published:** 2021-03-24

**Authors:** Junhui Jeong, Kyuin Lee, Hyun Seung Choi

**Affiliations:** Department of Otorhinolaryngology, National Health Insurance Service Ilsan Hospital, Goyang, Republic of Korea

## Abstract

Nevus is a benign melanocytic neoplasm and the most common type of skin tumor. It may occur anywhere on the skin, but it is rare in the external auditory canal (EAC). We present a case of melanocytic nevus in the EAC with keratin accumulation. In microscopic surgery, the mass was excised completely, and the wax and keratin material medial portion of the EAC behind the mass was removed. In this patient, a melanocytic nevus in the EAC caused symptoms of hearing loss and wax and keratin buildup. For melanocytic nevus in the EAC, excision and pathologic confirmation should be performed if there are symptoms or when malignant transformation is suspected.

## 1. Introduction

Nevus is a benign melanocytic neoplasm and the most common type of skin tumor [1–4]. It may occur anywhere on the skin, but it is rare in the external auditory canal (EAC) [1, 2, 4–6]. We present a case of melanocytic nevus in the EAC with keratin accumulation.

## 2. Case Presentation

**Figure 1:**
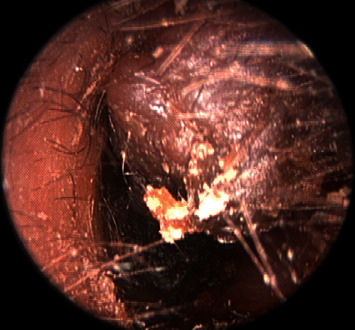
A brownish, firm, papillomatous, painless mass in the cartilaginous posterior portion of the left external auditory canal.

**Figure 2:**
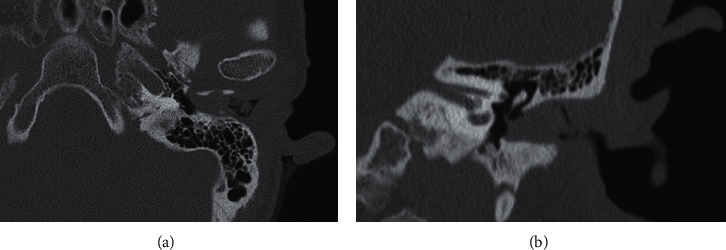
Temporal bone computed tomography revealing a 0.5 × 0.5 cm low-density mass in the cartilaginous portion of the left external auditory canal and low-density material with adjacent bony erosion medial to the mass: (a) axial view; (b) coronal view.

A 57-year-old woman presented with hearing loss on the left side for several days. She reported experiencing ear fullness on the left for several months prior. On physical examination, a brownish, firm, papillomatous, painless mass was observed in the cartilaginous posterior portion of the left EAC (Figure 1). Wax and desquamated keratin material were also observed in the medial portion of the EAC behind the mass. On temporal bone computed tomography, a 0.5 × 0.5 cm low-density mass was observed in the cartilaginous portion of the left EAC, and a low-density material with adjacent bony erosion was observed medial to the mass (Figure 2). Considering the possibility of a tumorous lesion including EAC cholesteatoma, complete excision was planned under local anesthesia.

**Figure 3:**
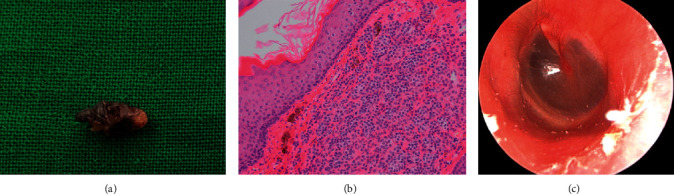
Surgical specimen of the 0.5 × 0.5 cm, brownish, firm, papillomatous mass (a). Histopathologic image showing dermal nests and cords of nevus cells with no junctional activity (hematoxylin-eosin, ×200) (b). Postoperative endoscopic view of the left external auditory canal showing no recurrence and no bony erosion (c).

In microscopic surgery, the 0.5 × 0.5 cm, brownish, firm, papillomatous mass was excised completely (Figure 3(a)), and the wax and keratin material medial portion of the EAC behind the mass was removed. The symptoms of hearing loss and ear fullness improved immediately after surgery. On histopathologic examination, nests of nevus cells with papillomatous proliferation were observed in the dermis. Dermal nests and cords of nevus cells showed no junctional activity (Figure 3(b)). Intradermal melanocytic nevus was thus diagnosed. One year and one month later, there was no recurrence and no bony erosion (Figure 3(c)).

## 3. Discussion

The pathogenesis of melanocytic nevus is thought to be junctional nests proliferating within the epidermis, with nevus cells migrating into the papillary dermis resulting in clusters of cells [3]. Factors related to melanocytic nevus include age, race, genetics, and environmental factors such as excessive exposure to sunlight [3, 4].

Melanocytic nevi are classified into acquired and congenital groups according to the time of appearance. Most cases are acquired and appear in childhood or puberty with a peak in the fourth decade [3]. Several studies of melanocytic nevus in the EAC have reported a predominance in females [3, 5, 7].

Melanocytic nevus can be divided into five different types: flat, slightly elevated, papillomatous, dome-shaped, and pedunculated [1, 2, 4–6]. The first three types are always pigmented, and the latter two types may or may not be pigmented [2]. It can also be divided according to the site of the nevus cell cluster; clusters of nevus cells in the epidermis can be classified as a junctional nevus, in the dermis as an intradermal nevus, and in both areas as a compound nevus [1–3, 5, 6]. Nevus cells evolve from the epidermis into the dermis, so the junctional nevus is common in children, and intradermal nevus is common in adults [5]. Most dome-shaped and papillomatous nevi are found in intradermal nevi [3].

Most melanocytic nevi are benign, and treatment is not necessary if they are asymptomatic [3, 5]. Surgical excision can be considered to alleviate symptoms or in cases of irregular borders or sudden increases in size [5]. There have been no reports of recurrence after excision [7].

Melanocytic nevus in the EAC could occlude the EAC, and wax and desquamated keratin could accumulate in the medial to the EAC mass. Impaired migration function of the EAC wall due to melanocytic nevus in the EAC could cause EAC cholesteatoma. Intradermal nevus with secondary EAC cholesteatoma has been reported previously [5]. Any large mass that occludes the EAC could cause EAC cholesteatoma. If the mass is pigmented, dome-shaped, or papillomatous, melanocytic nevus should be considered. EAC cholesteatoma or keratosis obturans can be accompanied by large-sized melanocytic nevus.

Melanocytic nevus should be differentiated from freckle, seborrheic keratosis, senile keratosis, pigmented actinic keratosis, common warts, pigmented fibrous histiocytoma, squamous papilloma, dermatofibroma, dysplastic nevus, squamous cell carcinoma, and malignant melanoma [2–5, 7]. Malignant melanoma, however, is the most important differential diagnosis [3]. Melanoma progresses over time, whereas melanocytic nevus grows to a point, stabilizes, and then involutes [2].

Some authors suggest that all EAC nevi should be removed, whereas others only recommend removal of symptomatic masses. Although there have been no reports of transformation of EAC melanocytic nevus into malignant melanoma, a large mass in the EAC could cause symptoms of hearing loss and EAC cholesteatoma, so large EAC melanocytic nevus should be removed, and pathologic confirmation should be performed. In particular, in cases of accumulated keratin and wax behind the mass, the mass and accumulated material should be removed to prevent the progression of EAC cholesteatoma.

In this patient, a melanocytic nevus in the EAC caused symptoms of hearing loss and wax and keratin buildup. Hearing loss was improved immediately after removal of the mass and accumulated keratin and wax. For melanocytic nevus in the EAC, excision and pathologic confirmation should be performed if there are symptoms or when malignant transformation is suspected.

## Data Availability

Data sharing is not applicable to this article as no datasets were generated or analysed during the current study.
